# *Arabidopsis* RRP6L1 and RRP6L2 Function in *FLOWERING LOCUS C* Silencing *via* Regulation of Antisense RNA Synthesis

**DOI:** 10.1371/journal.pgen.1004612

**Published:** 2014-09-11

**Authors:** Jun-Hye Shin, Julia A. Chekanova

**Affiliations:** School of Biological Sciences, University of Missouri - Kansas City, Kansas City, Missouri, United States of America; University of Massachusetts at Amherst, United States of America

## Abstract

The exosome complex functions in RNA metabolism and transcriptional gene silencing. Here, we report that mutations of two *Arabidopsis* genes encoding nuclear exosome components *AtRRP6L1* and *AtRRP6L2*, cause de-repression of the main flowering repressor *FLOWERING LOCUS C* (*FLC*) and thus delay flowering in early-flowering *Arabidopsis* ecotypes. *AtRRP6L* mutations affect the expression of known *FLC* regulatory antisense (AS) RNAs AS I and II, and cause an increase in Histone3 K4 trimethylation (H3K4me3) at *FLC*. AtRRP6L1 and AtRRP6L2 function redundantly in regulation of *FLC* and also act independently of the exosome core complex. Moreover, we discovered a novel, long non-coding, non-polyadenylated antisense transcript (ASL, for Antisense Long) originating from the *FLC* locus in wild type plants. The AtRRP6L proteins function as the main regulators of ASL synthesis, as these mutants show little or no ASL transcript. Unlike ASI/II, ASL associates with H3K27me3 regions of *FLC*, suggesting that it could function in the maintenance of H3K27 trimethylation during vegetative growth. AtRRP6L mutations also affect H3K27me3 levels and nucleosome density at the *FLC* locus. Furthermore, AtRRP6L1 physically associates with the ASL transcript and directly interacts with the *FLC* locus. We propose that AtRRP6L proteins participate in the maintenance of H3K27me3 at *FLC via* regulating ASL. Furthermore, AtRRP6Ls might participate in multiple *FLC* silencing pathways by regulating diverse antisense RNAs derived from the *FLC* locus.

## Author Summary

*Arabidopsis* FLOWERING LOCUS C (FLC) delays flowering; therefore, repressing expression of *FLC* provides a critical mechanism to regulate flowering. This mechanism involves multiple levels of regulation, including genetic regulation by transcription factors, and epigenetic regulation by modifications of genomic DNA and histones at the *FLC* locus. This work examines the role of non-coding RNAs in the epigenetic regulation of *FLC*, finding that the different RNAs produced from the *FLC* locus may have different functions in altering the epigenetic landscape at the *FLC* locus, and revealing that AtRRP6L1 and AtRRP6L2, two components of the exosome, an RNA-processing complex, play roles in regulating these non-coding RNAs. Therefore, this work explores the complex ties between RNA processing, non-coding RNAs, and epigenetic regulation of *FLC*, a key repressor of flowering.

## Introduction

The regulation of gene silencing occurs at multiple levels and non-coding RNAs (ncRNAs) have emerged as important regulators of genome silencing at the transcriptional and posttranscriptional levels [1]. At the chromatin structure level, chromatin remodeling factors and DNA- and histone-modifying enzymes alter chromatin to control its structure and compaction, thus affecting silencing [2]. ncRNAs significantly contribute to the regulation of chromatin structure and play important roles in eukaryotic genomes by affecting the epigenetic architecture, including both establishment and maintenance of epigenetic marks. ncRNAs can initiate heterochromatin formation through the RNA interference (RNAi) pathway, or independently of RNAi, and through the RNA processing machinery [3], [4].

The exosome is an evolutionarily conserved complex of RNase-like and RNA binding proteins involved in 3′ to 5′ decay and processing of various RNA substrates [5]–[10]. The exosome complex plays an important role in regulating both coding and ncRNAs [11]. The nuclear and cytoplasmic forms of the eukaryotic exosome complex share ten common subunits [12]. In most organisms, all nine subunits of the exosome core complex are inactive, and enzymatic activities are provided by the tenth catalytic RRP44 subunit, a 3′-5′ hydrolytic exoribinuclease, which also has endonucleolytic activity [13].

The nuclear form of eukaryotic exosome also associates with the second catalytic RRP6 subunit, a substoichiometric, nuclear-specific, 3′ to 5′ exoribonuclease [14]–[16]. The RRP6 subunit has a number of unique functions in the exosome [14], and also additional functions not associated with the exosome core [17]–[19]. *Arabidopsis* has three possible functional homologs of RRP6: the nuclear proteins AtRRP6L1 and AtRRP6L2, and the cytoplasmic protein AtRRP6L3 [20]. When we purified the exosome complex from Arabidopsis, AtRRP6L proteins were not detected in our experiments, which is likely due to the fact that, as a substoichiometric subunit restricted to a nuclear form, it was underrepresented in our preparations [8]. Therefore, whether these *Arabidopsis* RRP6Ls physically associate with the exosome core remains to be tested.

The exosome complex broadly affects epigenetic silencing of heterochromatic and euchromatic loci by regulating a variety of ncRNAs [11], [21]–[24]. In fission yeast (*Schizosaccharomyces pombe*), the exosome acts in several different small RNA (smRNA) pathways to affect constitutive and facultative heterochromatin silencing, in either RNAi-dependent or RNAi-independent manners [4], [25]–[27]. The exosome also acts in gene silencing through RNA quantity and quality surveillance, and in collaboration with the 3′ termination machinery [21], [22], [28]–[30].

Our previous genome-wide survey revealed that many exosome targets in *Arabidopsis* correspond to ncRNAs, many originating from heterochromatic loci, suggesting that the exosome participates in various silencing pathways in *Arabidopsis* [8]. Our recent analysis of exosome functions in smRNA-mediated silencing of genes in *Arabidopsis* showed that the exosome has little effect on the smRNAs that function in the main silencing mechanisms, siRNA-dependent methylation of DNA (RdDM). Rather, we showed that the exosome associates physically with long, polyadenylated RNAs transcribed from the scaffold regions of several heterochromatic loci, and exosome defects affected the level of histone H3K9me2, an epigenetic mark that alters chromatin structure [31]. We also found that the *Arabidopsis rrp6* homologues AtRRP6L1 and AtRRP6L2 participate in these epigenetic mechanisms and may function redundantly [31]. With the exception of *AtCSL4*, the genes encoding the *Arabidopsis* exosome core complex subunits are essential for viability [8]. By contrast, unlike the core exosome subunits, the exosome nuclear catalytic subunit RRP6 and the *Arabidopsis* RRP6-Like proteins are not essential for viability [14], [20]; thus, the *rrp6* and *rrp6l* mutants provide tools to study the role of the exosome during development.

Epigenetic regulation by long non-coding RNAs (lncRNAs) and histone modifications plays a key role in controlling the expression of *Arabidopsis FLC (FLOWERING LOCUS C)*, which encodes a MADS-box transcription factor that suppresses flowering [32]–[35].

*FLC* regulation through chromatin modifications has been well studied [36], [37]. *FLC* expression requires H3K4 methylation, a permissive chromatin modification, on *FLC* chromatin, and H3K4 demethylation leads to *FLC* repression [37]–[39]. *FLC* silencing requires Polycomb complex 2 (PRC2) -deposited H3K27me3, a repressive chromatin modification [36], [40], [41]. Repressing the expression of *FLC* provides a central mechanism for both the vernalization pathway, which regulates flowering time in response to periods of prolonged cold, and the autonomous pathway, which regulates flowering independently of environmental signals [37].

The processing and metabolism of lncRNAs play a crucial role in *FLC* silencing, and different lncRNAs produced from the *FLC* locus may have distinct functions. For example, lncRNAs COOLAIR and COLDAIR participate in the epigenetic silencing of the *FLC* locus [40], [42]. The COOLAIR antisense transcript is produced from the *FLC* locus as two alternatively polyadenylated isoforms, AS I and AS II, and it was shown that the processing of AS I and II function in *FLC* epigenetic silencing by affecting H3K4 demethylation [37], [38], [42]. COOLAIR transcription does not appear to be required for vernalization, but it has been implicated in *FLC* repression early during cold treatment, possibly mediated by direct effects on the *FLC* promoter [37]. By contrast, the COLDAIR sense transcript is produced from within *FLC* intron 1, and plays a role in *FLC* silencing *via* recruitment of Polycomb repressing complex 2 (PRC2) to *FLC* during vernalization [40]. The establishment of H3K27 trimethylation to silence *FLC* in vernalization requires COLDAIR [40], but not AS I and AS II [41], [43]. Also, in the autonomous pathway, the proteins involved in 3′-end RNA processing act as the main factors in *FLC* silencing [38], [39], [44]–[46].

Here, we report that mutations of *AtRRP6L1* and *AtRRP6L2* result in delayed flowering in early-flowering *Arabidopsis* ecotypes that do not require vernalization for flowering. We found that AtRRP6L1 and AtRRP6L2 epigenetically regulate *FLC* silencing by regulating different antisense transcripts and modulating H3K4me3 and H3K27me3 histone modifications at the *FLC* locus. Moreover, we discovered a novel antisense transcript, termed Antisense Long (ASL), which originates from the *FLC* locus in wild type plants and is regulated by AtRRP6L1 and AtRRP6L2. Our study demonstrates that *Arabidopsis* RRP6L proteins play an important role in the regulation of genes expressed in specific developmental phases *via* participating in lncRNA-mediated epigenetic silencing.

## Results

### Mutations in *AtRRP6L1* and *AtRRP6L2* result in late flowering, caused by de-repression of *FLC* expression

We previously examined T-DNA mutations in *Arabidopsis RRP6L1* and *RRP6L2* and found that these mutations lead to de-repression of known heterochromatic loci and that RRP6L1 and 2 likely function redundantly in this process [31]. The T-DNA insertion allele of *AtRRP6L1* was isolated from the Wisconsin population of T-DNA mutants [31], in the Wasilevskaya (Ws) ecotype, and the T-DNA insertion allele of *AtRRP6L2* comes from the SALK collection, in the Columbia (Col-0) ecotype [31]. We previously used RT-PCR analysis to demonstrate that *rrp6l1-3* mutant is a null allele and *rrp6l2-3* is nearly null [31].

**Figure 1:**
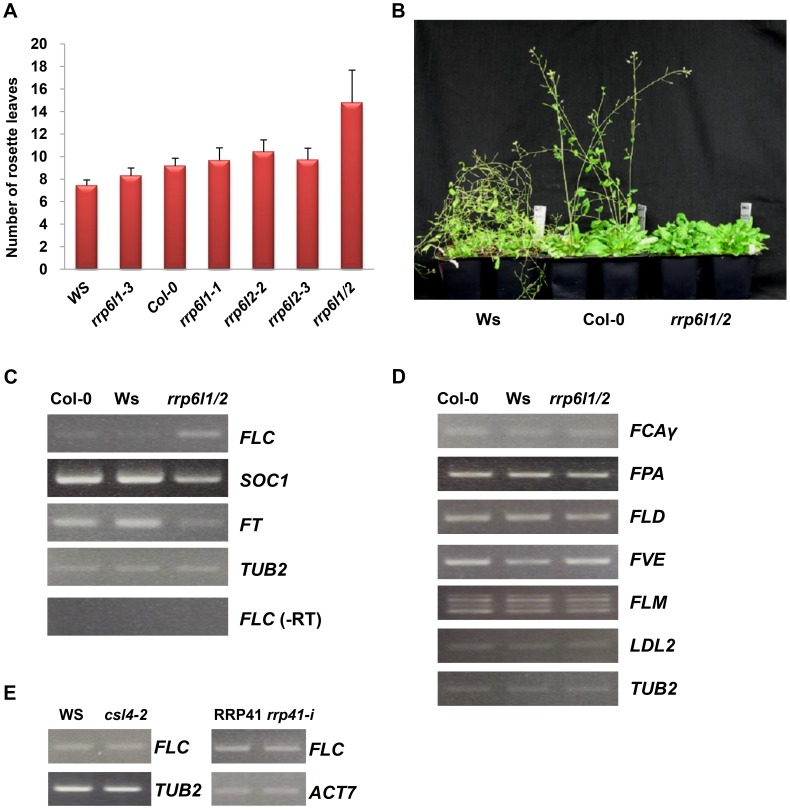
The *rrp6l1-3* and *rrp6l2-3* mutants affect flowering time and gene expression. (A) The late-flowering phenotype of *rrp6l1* and *rrp6l2* mutants grown under long day conditions. Flowering time was measured as rosette leaf number at bolting. To control for effects of ecotype, the phenotype of mutants was compared to wild-type plants of Col-0 and Ws ecotypes. Error bars represent standard deviation (SD). (B) The late-flowering phenotype of *rrp6l1-3 rrp6l2-3* mutants grown under short day conditions. 66-day-old plants are shown. (C) Effect of *rrp6l1-3* and *rrp6l2-3* mutations on the expression of the *FLC*, *SOC1* and *FT*. RT-PCR showed that the *rrp6l1-3 rrp6l2-3* double mutant (*rrp6l1/2*), has increased expression of *FLC* and decreased expression of *SOC1* and *FT*, which act downstream of *FLC*. (-RT) is the no reverse transcriptase control. (D) The expression of *FCA*, *FPA*, *FLD*, *FVE*, *FLM*, and *LDL2*, genes involved in regulation of flowering time in the autonomous flowering pathway, is not affected in *rrp6l1/2* mutants. (E) *FLC* expression is not affected in *AtCSL4*-2 T-DNA mutant and *RRP41 iRNAi* line. RRP41 corresponds to the *iRNAi* line grown without estradiol, and *rrp41-i* corresponds to line grown on estradiol-containing medium, to induce the RNAi-mediated knockdown of *RRP41*. *TUBULIN 2* and *ACTIN 7* were used as loading controls.

To control for the ecotype, we examined the phenotypes of *rrp6l1-3*, *rrp6l2-3*, and *rrp6l1-3 rrp6l2-3* double mutants (hereafter *rrp6l1/2*) and compared them to wild-type plants of Col-0 and Ws ecotypes. When we examined the phenotype of different *rrp6l1* and *rrp6l2* alleles, we found that these single mutants did not show significant phenotypic alterations, although they exhibited a mild delay in flowering, as measured by leaf number at bolting (Fig. 1A). By contrast, the late-flowering phenotype becomes pronounced in *rrp6l1/2* double mutants grown under long day conditions (Fig. 1A) and becomes very severe in plants grown under short day conditions (Fig. 1B). These findings indicate that AtRRP6L1 and AtRRP6L2 likely have redundant functions in the pathways activating flowering. The flowering defects in plants grown under short and long day conditions suggest that AtRRP6L1 and AtRRP6L2 function in the regulation of flowering through a pathway different from the photoperiod pathway, which promotes flowering in response to day length. The vernalization and autonomous pathways promote flowering through repression of *FLC* expression. During vernalization, cold temperatures repress *FLC* expression, and FRIGIDA (FRI) activates *FLC* expression [35]. Ecotypes that lack functional alleles of *FRI*, such as the early-flowering accessions Col-0 and Ws ecotypes used in this study, do not require vernalization for flowering. Thus, our data suggest that AtRRP6L1and AtRRP6L2 could be involved in regulation of *FLC* expression through the autonomous flowering pathway.

To examine the roles of AtRRP6L1and AtRRP6L2 in regulation of *FLC* expression, we examined whether mutations of *AtRRP6Ls* affect the expression of *FLC*. We observed no change in *FLC* expression in the *rrp6l1-3* and *rrp6l2-3* single mutants, consistent with the degree of the observed phenotypic alterations, and we confirmed this observation with additional *AtRRP6L1* and *AtRRP6L2* T-DNA alleles (Fig. S1B). By contrast, we observed an increase in the levels of *FLC* transcript in the *rrp6l1/2* double mutant relative to wild-type plants (Fig. 1C). These results suggest that AtRRP6L1 and AtRRP6L2 affect *FLC* expression and function redundantly in this process. To make sure that this phenotype does not result from transgressive segregation, we constructed *AtRRP6Ls* mutants using different *AtRRP6L* alleles isolated from the same Col-0 background and confirmed that this mutant combination causes a similar delay in flowering and derepression of *FLC* (Fig. S1A).

**Figure 2:**
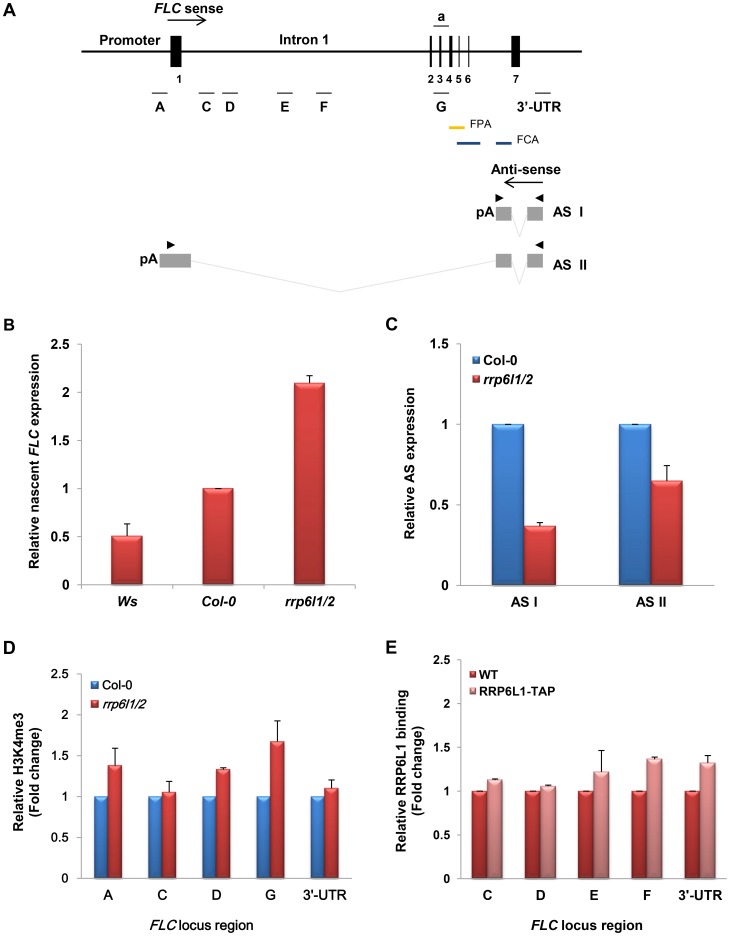
The effect of the *rrp6l1-3 and rrp6l2-3* mutations on expression of *FLC* sense and antisense transcripts, and on H3K4 methylation. (A) The diagram of the *FLC* gene based on analysis of the transcription unit [38] Vertical bars and numbers denote the exons of the *FLC* sense transcript. The transcription start site of the *FLC* sense mRNA is indicated by an arrow. Two antisense transcripts, AS I (proximal) and AS II (distal), are depicted below the *FLC* diagram. Antisense transcripts are alternatively polyadenylated, with a proximal poly(A) site in sense intron 6 and a distal poly(A) site in the sense promoter region. Grey boxes correspond to AS I and II exons, and grey lines correspond to the spliced regions of the antisense RNAs. Horizontal bars (letters a, A to G and 3′-untranslated region, 3′UTR), correspond to the *FLC* regions used in qRT-PCR and ChIP. Arrowheads correspond to position of primers used for RT-PCR amplification of AS I and II. Blue bars correspond FCA binding region, yellow bar correspond to the FPA binding region. (B) Nascent *FLC* expression significantly increased in *rrp6l1/2* mutants. Expression of *FLC* was measured by qRT-PCR and is shown relative to *FLC* expression in Col-0 and Ws wild type plants; (C) Expression of AS I and AS II transcripts in *rrp6l1/2* mutants. Expression of AS I and AS II in *rrp6l1/2* was compared to their expression in Col-0 wild type plants by qRT-PCR. The antisense transcript levels were normalized by total antisense transcript as described previously [38]. Error bars represent standard deviation (SD). (D) Effect of *rrp6l1-3* and *rrp6l2-3* mutations on the level of H3K4me3 examined by ChIP using antibodies against H3K4me3 in the various regions of *FLC*. The level of H3K4me3 in *rrp6l1/2* mutants was normalized to the level of H3K4me3 in wild type Col-0 plants. (E) AtRRP6L1 protein physically interacts with the *FLC* locus. ChIP assays were done using the *rrp6l1-3* mutant complemented by a functional *AtRRP6L1-TAP* transgene. AtRRP6L1-TAP recruitment was normalized to wild type Ws, the background of the *rrp6l1-3* mutant. The error bars in ChIP experiments represent the standard error of the mean and correspond to the difference between 2 biological replicates.

FLC acts as a dosage-dependent floral repressor [47]. The exosome complex and RRP6 subunits affect the processing and turnover of various RNAs, and thus regulate RNA quality and quantity [5], [8], [11]. To find out whether the *FLC* transcript, which increased in abundance in *rrp6l1/2* mutants, corresponds to a functional transcript rather than nonfunctional byproduct, we examined the expression of the flowering genes *SUPPRESSOR OF CONSTANS OVEREXPRESSION 1* (*SOC1*) and *FLOWERING LOCUS T* (*FT*), which act downstream of *FLC*. We found that the *rrp6l1/2* mutants showed lower levels of both *SOC1* and *FT* transcripts (Fig. 1C), indicating that the increased level of *FLC* transcript in *rrp6l1/2* mutants corresponds to a functional *FLC* transcript, and the increase in *FLC* expression enhances the repression of the downstream genes. In addition to increased levels of *FLC* transcript, we also detected increased amounts of the unspliced *FLC* RNA in *rrp6l1/2* mutants (Fig. 2B).

The autonomous pathway constitutively represses *FLC*, independent of environmental inputs [35]. To find out whether AtRRP6L1 and AtRRP6L2 affect *FLC* expression directly or by regulating the expression of the upstream genes that silence *FLC* in the autonomous pathway, we examined the expression of upstream genes in the *AtRRP6L1* and *AtRRP6L2* mutants. We found that the *rrp6l1/2* plants showed no changes in expression of the genes that act upstream of *FLC* (Fig. 1D). These data imply that the de-repression of *FLC* observed in *AtRRP6L* mutants results from a direct effect of AtRRP6Ls on the expression of *FLC*, not from regulation of the genes acting upstream of *FLC*.

To test whether the effect of *rrp6l1/2* on *FLC* expression requires the core exosome complex, we next examined *AtCSL4-2* and *AtRRP41*, which encode core exosome complex subunits. We did not observe de-repression of *FLC* transcription in an *AtRRP41* inducible RNAi line or in *AtCSL4-2* T-DNA insertion mutants (Fig. 1E), suggesting that AtRRP6L1 and AtRRP6L2 likely function independently of the exosome in the regulation of *FLC* expression.

### Mutations of *AtRRP6Ls* affect expression of the antisense transcripts that regulate expression of *FLC*

Antisense transcripts regulate *FLC* silencing and antisense expression appears to independently intersect with both the vernalization and autonomous pathways to repress *FLC* expression [37]. During the vegetative phase, the *FLC* locus produces two alternatively spliced, polyadenylated regulatory antisense (AS) transcripts, AS I and AS II [37] (Fig. 2A). Targeted 3′ end processing of these antisense transcripts affects the recruitment of histone chromatin remodelers to the locus, which results in reduced *FLC* transcription [38], [39], [46]. Therefore, we investigated whether the *rrp6l1/2* mutants showed changes in the ratio of 3′ end processing and polyadenylation of these antisense transcripts. We found that, compared to wild type plants, the *rrp6l1* or *rrp6l2* single mutants, and the *rrp6l1/2* double mutants had lower levels of processed AS I and II transcripts (Fig. 2C and Fig. S1C); also, the *rrp6l1/2* plants showed reduced levels of AS I and II, consistent with the stronger phenotype of the *rrp6l1/2* mutants (Fig. 1A and S1C). Interestingly, the pattern of down-regulation of AS I and II transcripts in *rrp6l1/2* mutants was similar to the pattern observed in the mutants of cleavage stimulation factors *CstF64* and *CstF77*, components of the cleavage polyadenylation machinery required for the 3′-end processing of AS I and II transcripts [38]. RRP6 plays an important role in formation of the 3′ ends of many RNAs in yeast and humans [14], [48], [49], and in budding yeast, also participates in the regulation of antisense transcripts derived from the *PHO84* locus [21], [50]. Therefore, it is possible that AtRRP6L1 and AtRRP6L2 proteins, along with CstF, could participate in the 3′ end processing of both antisense transcripts. To further investigate the relationship between CstF64 and AtRRP6Ls, we attempted to construct a triple *rrp6l1-3 rrp6l2-3 cstf64-2* mutant; however, since *cstf64-2* mutants are sterile, we could not obtain the triple homozygous mutant. Taken together, our data suggest that AtRRP6L proteins could negatively regulate *FLC* expression by affecting the expression of the regulatory antisense transcripts.

### Chromatin structure of the *FLC* locus is altered in *rrp6l1/2* plants

Methylated H3K4 marks active chromatin states and the antisense transcripts synthesized from *FLC* may function in *FLC* silencing by recruiting chromatin remodeling factors that drive H3K4 demethylation [38], [39]. To investigate whether the decrease in the antisense transcripts in the *rrp6l1/2* mutants leads to changes in histone modifications at the *FLC* locus, we used chromatin immunoprecipitation (ChIP) to analyze the levels of H3K4me3 at various regions of the *FLC* locus (Fig. 2A). We found that the *rrp6l1/2* mutant had significantly increased levels of H3K4me3 along the entire length of *FLC*, compared with wild type (Fig. 2D). These data suggest that the decrease in the level of antisense transcripts in *rrp6l1/2* mutants might lead to the decreased recruitment of chromatin remodeling factors required for H3K4 demethylation, thereby regulating the accessibility of the transcription machinery to the locus, and in turn leading to *FLC* de-silencing, similar to previously reported observations [38].

### AtRRP6L1 directly interacts with the *FLC* locus

The expression of *FLC* sense and antisense transcripts together with the increased level of H3K4 trimethylation in *rrp6l1/2* mutants suggested that AtRRP6s could participate in *FLC* transcriptional silencing by affecting the chromatin structure at the *FLC* locus. Therefore, we asked whether AtRRP6L proteins can directly interact with the *FLC* locus to participate in the silencing pathway. To address this question, we constructed transgenic *rrp6l1-3* lines that were complemented by a wild type copy of *AtRRP6L1* fused with the TAP-tag for affinity purification, AtRRP6L1-TAP (see Methods).

We then used ChIP on these lines to examine the association of AtRRP6L1 protein with several regions of *FLC* (Fig. 2A). ChIP showed a modest enrichment of AtRRP6L1 protein in the regions corresponding to the 3′-UTR and intron 1 of *FLC* (Fig. 2E). The AtRRP6L1 binding region within intron 1 appears to be further downstream of the 3′-end of the AS I transcript (with respect to the direction of AS I and II transcription), implying that AtRRP6L1 may bind in this region to process a longer antisense precursor transcript. The AtRRP6L1 binding regions do not overlap with the regions reported to be bound by FPA and FCA proteins, RNA-binding 3′-end processing factors required for the processing of the AS I transcript [39], [46]. However, FPA binds to the region between exon 4 and 5 (Fig. 2A), which is also downstream of the 3′-end region of AS I [46]; thus, AtRRP6L1 and FPA have somewhat similar, but not identical, binding patterns (Fig. 2A and E). The association of AtRRP6L1 protein with a larger region of the *FLC* locus (downstream of the 3′end of AS I) also suggests that AtRRP6L proteins might participate in the processing of different types of antisense RNAs, in addition to the known antisense transcripts derived from the *FLC* locus. Alternatively, AtRRP6L could also participate in co-transcriptional regulation of antisense transcription by binding to nascent antisense transcripts. Also, the level of AtRRP6L1 enrichment at the *FLC* locus was relatively modest (Fig. 2E). Thus, we cannot rule out the possibility that AtRRP6L proteins could associate with the locus by binding other protein and RNA complexes that physically interact with the locus.

### A novel antisense transcript in the *FLC* locus

**Figure 3:**
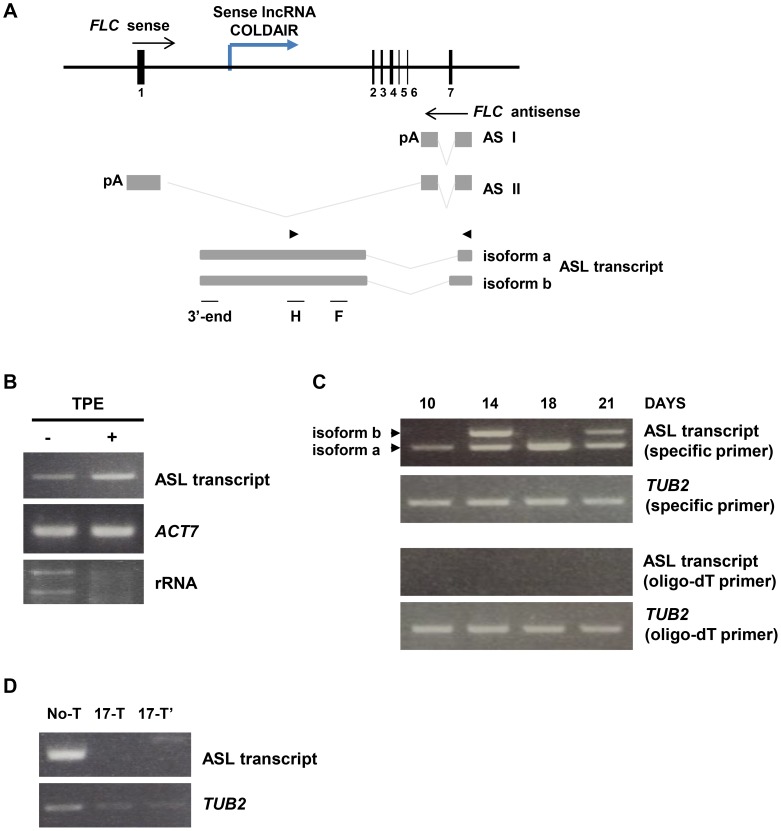
Characterization of the ASL transcript. (A) The diagram of the *FLC* gene and ASL RNA produced from the *FLC* locus in wild type plants. Gray boxes depict antisense transcript exons and gray lines indicate spliced regions. Alternatively spliced antisense transcript isoforms found by tiling RT-PCR. Arrowheads indicate position of primers used for RT-PCR amplification of ASL. F, H and 3′-end indicate that the regions used for RT-PCR amplification after RNA-IP (in Fig. 5A and B). The blue line depicts the COLDAIR transcript. (B) The ASL transcript is capped at the 5′end. RNA samples of Col-0 plants were treated with Terminator 5′-Phosphate-Dependent Exonuclease (TPE), which degrades uncapped RNA. (−) corresponds to the RNA sample before TPE treatment and (+) corresponds to the TPE treated sample. *ACTIN 7* and rRNA were used as capped and un-capped controls for TPE treatment, respectively. (C) Expression of ASL in Col-0 plants at 10, 14, 18 and 21 days after germination of vegetative phase. Arrowheads indicate the two alternatively spliced isoforms of ASL. cDNA was synthesized using either antisense RNA specific primers or oligo-dT primers. *TUBULIN 2* was used as an internal control and was amplified from the sample reverse transcribed with either tubulin 2-specific primers (second row) or the oligo-dT primers (bottom row). (D) α-amanitin treatment of Col-0 (11 days seedlings). No-T corresponds to non-treated plants, and 17-T corresponds to plants treated with α-amanitin for 17 hours. 17-T and 17-T′ indicate independent biological replicates.

When we were examining the pattern of known antisense transcripts produced from the *FLC* locus, we observed the presence of a different antisense transcript in wild type plants, but not in *rrp6l1/2* plants. Therefore, we set out to investigate the features of this antisense transcript by tiling RT-PCR using a set of primers that cover the entire *FLC* locus (Fig. S2A). We found that the transcript is a novel antisense RNA of over 2000 nucleotides in length. The sequence of this antisense RNA, which we termed ASL (Antisense Long), corresponds to intron 1 and the 3′-UTR region of the sense *FLC* transcript (Fig. 3A). Interestingly, 5′ region of the ASL transcript overlaps with the 5′ region of the AS I and II transcripts. Sequencing of ASL revealed that it has two different isoforms, 2,236 nt and 2,536 nt (ASLa and b, respectively), produced by alternative splicing (Fig. 3A). Moreover, ASL spans intron 1, an important region for maintenance of *FLC* silencing [51]. Notably, ASL also overlaps with the COLDAIR lncRNA, which is transcribed within intron 1 in the sense direction during vernalization [40] (Fig. 3A).

To determine whether the ASL RNA has a 5′ cap, we used Terminator 5′-Phosphate-Dependent Exonuclease (TPE), which degrades uncapped RNA. We found that TPE treatment did not affect ASL levels, indicating that ASL has a 5′ cap (Fig. 3B). Next, we examined the 3′ end of ASL by performing cDNA synthesis primed by either sequence specific primer or oligo-dT primers. To our surprise, we found that ASL is not polyadenylated, as we detected ASL only from the cDNA primed by specific primers, not by oligo-dT (Fig. 3C).

In plants, the RNA polymerases RNA Pol IV and Pol V [52] participate in gene silencing through smRNA-mediated mechanisms [53]–[55]. To investigate which RNA polymerase synthesizes ASL, we treated plants with α-amanitin, an inhibitor of RNA Pol II, and then used RT-PCR to examine ASL levels. We did not detect ASL in plants treated with α-amanitin (Fig. 3D), implying that RNA Pol II synthesizes ASL. To confirm this, we also examined the presence of ASL in *nrpd1* (Pol IV) and *nrpe1* (Pol V) mutants; we detected ASL in these mutants, indicating that Pol IV and V do not affect ASL, although *nrpd1* mutants showed a minor change in ASL levels (Fig. S2B).

Taken together, our data indicate that ASL is capped, synthesized by RNA Pol II and non-polyadenylated, and also suggest that it is distinct from the known antisense transcripts originating from the *FLC* locus. The tiling RT-PCR analysis indicates that the same promoter produces ASL, AS I, and AS II. Thus, it is possible that the ASL transcript could function differently from AS I and II in the silencing of *FLC*. Indeed, different antisense RNAs transcribed from same promoter of the human pseudogene *PTENpg1* have different functions in transcriptional and post-transcriptional silencing of the tumor suppressor gene *PTEN* [56].

### AtRRP6L1 and AtRRP6L2 regulate ASL levels

**Figure 4:**
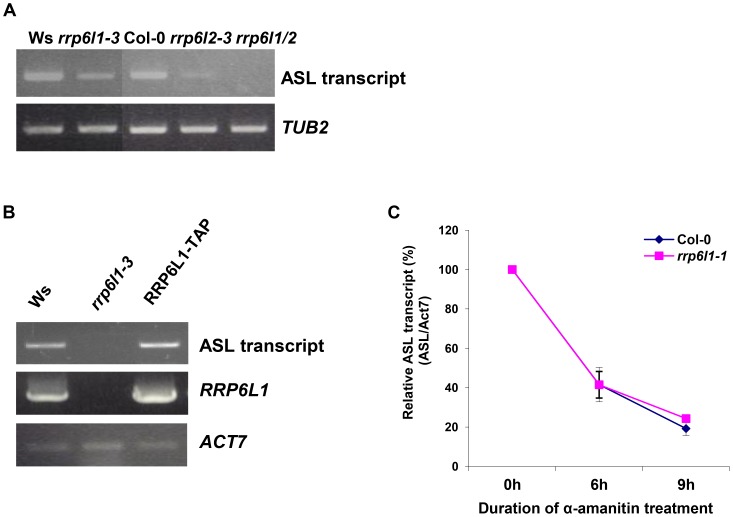
The level of ASL transcripts is decreased in *rrp6l* mutants. (A) Expression of ASL RNA in *rrp6l1-3*, *rrp6l2-3* and *rrp6l1/2* double mutants. (B) The level of ASL transcript expression is restored in the *rrp6l1-3* mutant complemented by functional *AtRRP6L1-TAP* transgene. *ACTIN 7* was used as a loading control. Transgenic plants are in Col-0 ecotype. (C) Decay rate of ASL transcript in *rrp6l1-1* mutants. α-amanitin treatment was performed for 0, 6 and 9 hours. The expression of antisense transcript was normalized to *ACTIN 7*.

Next, we investigated how the AtRRP6Ls participate in the regulation of ASL expression. We found that the level of ASL transcript decreased in *rrp6l1-3* and *rrp6l2-3* single mutants (Fig. 4A and Fig. S2C). Moreover, we detected little or no ASL transcript in *rrp6l1/2* double mutants (Fig. 4A), indicating that AtRRP6L proteins function as the main factors regulating the levels of the ASL transcript. Consistent with AtRRP6L functions in regulation of ASL expression, we observed that the level of the ASL transcript recovered to wild type levels in *rrp6l1-3* mutant complemented by the wild type copy of *AtRRP6L1-TAP* (Fig. 4B). These data suggest that both AtRRP6L1 and AtRRP6L2 directly regulate the expression of ASL and are the main factors in this process.

RRP6 is a 3′ - 5′ exoribonuclease and RRP6 defects usually result in abnormal accumulation of various RNAs due to failure to degrade or process them [21], [49], [50], [57]. We next asked how AtRRP6Ls could regulate ASL levels. The *rrp6l1/2* mutants showed nearly undetectably low levels of ASL. We reasoned that, if AtRRP6Ls regulate the stability of the ASL transcript, then we would observe a difference in ASL decay rate in *AtRRP6L* single mutants. Therefore, we used the *rrp6l1-1* single mutant and conducted an α-amanitin chase to compare the rates of ASL transcript decay in *rrp6l1-1* and wild type plants. We found that the *rrp6l1-1* mutant and wild type had similar rates of ASL transcript decay (Fig. 4C), suggesting that AtRRP6L proteins do not directly participate in the degradation of the ASL transcript but rather affect its production or biogenesis.

### AtRRP6L1 interacts with the ASL transcript, which physically associates with H3K27me3 regions of *FLC*

**Figure 5:**
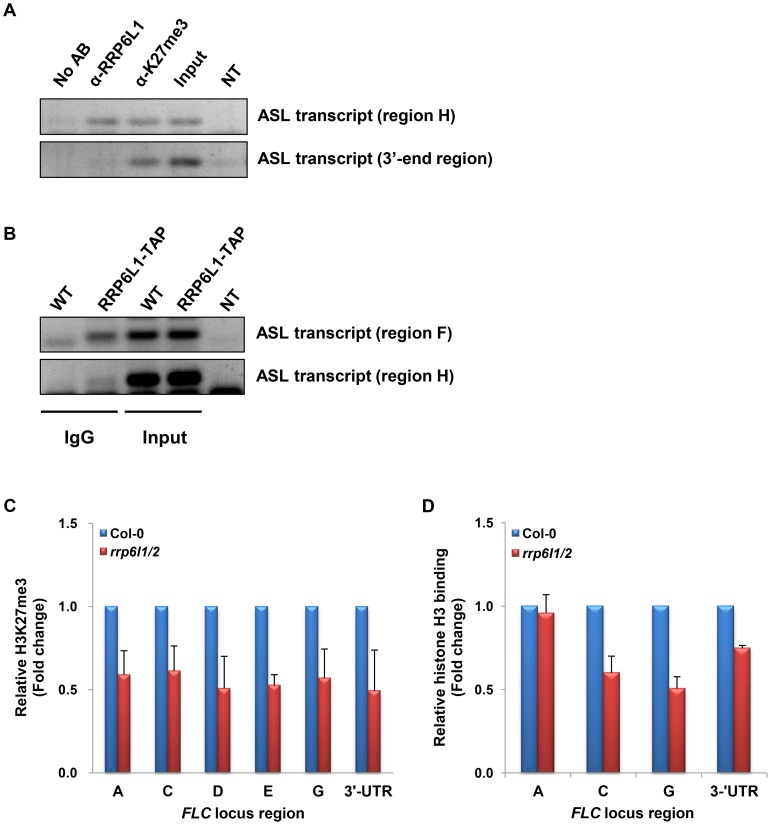
ASL transcript associates with AtRRP6L1 protein and H3K27 trimethylated regions and *rrp6l1/2* mutants have decreased H3K27me3 and nucleosome density. (A) ASL transcript directly associates with AtRRP6L1 protein and H3K27me3 regions of *FLC*. RNA-IP was performed using anti-AtRRP6L1 and anti-H3K27me3 antibodies to precipitate ASL RNA from wild type Col-0 plants. The regions used in RT-PCR (region H and the 3′end of ASL) are shown on Fig. 3A. No AB indicates no antibodies and is the negative control for RNA-IP. NT indicates no template and is the negative control for RT-PCR. (B) ASL transcript directly associates with AtRRP6L1 in *rrp6l1-3* mutants complemented by functional AtRRP6L1-TAP transgene. Transgenic plants are in the Ws ecotype and Ws was used as negative control for RNA-IP. RNA-IP was performed using IgG antibodies to co-precipitate ASL RNA with RP6L1-TAP from wild type plants complemented with a transgene expressing *RRP6L1-TAP*. NT indicates no template and is the negative control for RT-PCR. (C) The level of H3K27me3 is decreased in *rrp6l1/2* mutants. ChIP assays were performed using H3K27me3 antibodies. The level of H3K27me3 in *rrp6l1/2* mutants was plotted relative to the level of H3K27me3 in Col-0 plants. The error bars in ChIP experiments represent the standard error of the mean and correspond to the difference between 2 biological replicates. (D) Nucleosome density is decreased in *rrp6l1/2* mutants. Mnase-ChIP assays were performed using histone H3 antibodies. Nucleosomal density in *rrp6l1/2* mutants was plotted relative to the level of nucleosomal density in Col-0 plants. The error bars in ChIP experiments represent the standard error of the mean and correspond to the difference between 2 biological replicates.

To find out whether AtRRP6L1 could play a direct role in the expression of ASL, we examined if AtRRP6L1 protein physically associates with the ASL transcript. To this end, we conducted RNA immunoprecipitation (RNA-IP) in wild type plants using antibodies against AtRRP6L1 protein (Fig. 5A). The RNA-IP showed that AtRRP6L1 protein physically associates with the ASL transcript (Fig. 5A). We also obtained identical results from RNA-IP in the *rrp6l1-3* mutant complemented with *AtRRP6L1-TAP* (Fig. 5B). Together these results indicate that AtRRP6L1 protein likely participates directly in the regulation of ASL transcript levels.

We then hypothesized that the ASL RNA may play a role distinct from that of AS I and II. Histone remodeling factors affect *FLC* silencing and several lncRNAs affect H3K4 demethylation and H3K27 trimethylation [41]. The sense lncRNA COLDAIR participates in recruiting PRC2 and is necessary for the establishment of H3K27 trimethylation during vernalization [40], but this does not require AS I and II [41], [43]. However, H3K4 demethylation in the autonomous pathway does require AS I and II [37]–[39]. COLDAIR may also contribute to the maintenance of H3K27me3 during vegetative growth. In addition, the H3K27me3-binding protein LHP1 functions in the maintenance of H3K27me3 during the vegetative phase in actively dividing cells, suggesting that H3K27me3 maintenance could be important for *FLC* silencing during the vegetative phase after H3K27me3 has been established [58].

To examine whether ASL has a role distinct from that of AS I and II, we examined the level of the repressive histone mark, H3K27me3 at the *FLC* locus in *rrp6l1/2* mutants. To our surprise, we observed that the *rrp6l1/2* mutants showed significantly decreased levels of H3K27me3 along the entire *FLC* locus (Fig. 5C). The *rrp6l1-3* single mutant also showed a mild decrease in the level of H3K27me3 (Fig. S1D). This observation is consistent with the very mild phenotype and the decreased level of the ASL transcript observed in *rrp6l1* single mutants (Fig. 1A, 4A and S2C). Together, our data indicate that the knock-out of both *AtRRP6L1* and *AtRRP6L2* affected the levels of both H3K4me3 and H3K27me3 in the *FLC* locus (Fig. 3B). This is in contrast to AS I and II, which affect only the levels of H3K4me3 at *FLC*, at least in vernalization pathway [37], [41], [43].

The level of H3K27me3 correlates with the nucleosomal density [59], [60]. To examine whether the reduction in the level of H3K27me3 in *rrp6l/2* mutants affects nucleosome positioning, we performed Micrococcal Nuclease (MNase)-ChIP assays using anti-H3 antibodies. MNase degrades nucleosome-free regions, allowing the estimation of nucleosome density. We found a lower nucleosomal density at the *FLC* locus in *rrp6l1/2* mutants (Fig. 5D). These data indicate that the reduction of H3K27me3 levels observed in the *rrp6l1/2* mutants could also result in relaxation of the chromatin state, which then allows factors involved in *FLC* transcription to gain easier access to the locus. Taken together, our results suggest that the regulation of the ASL transcript by AtRRP6L proteins may contribute to the maintenance of H3K27me3 at the *FLC* locus, which in turn contributes to the compact chromatin structure of the locus.

The *rrp6l1/2* mutations lead to a decrease of H3K27me3 levels and also affect the nucleosome density at the *FLC* locus (Fig. 5C and D). Therefore, we asked whether the ASL transcript could be directly involved in H3K27 trimethylation, a role similar to that played by the COLDAIR lncRNA. To answer this question, we performed RNA-IP using antibodies against H3K27me3. We found that the ASL transcript physically associates with H3K27me3 regions (Fig. 5A). Taken together, our data suggest that the ASL transcript could function in the maintenance of H3K27 trimethylation during the vegetative phase.

## Discussion

Here, we report that AtRRP6L1 and AtRRP6L2, the homologues of rrp6 subunits of the exosome in other organisms, negatively regulate the expression of *FLC* to promote flowering in *Arabidopsis*, and act redundantly in this process. This finding provides the first evidence that plant RRP6 proteins participate in the regulation of a specific developmental process. Our data indicate that AtRRP6L proteins play a role in the regulation of antisense RNA production and the chromatin landscape of the *FLC* locus. Moreover, we identified a novel antisense transcript produced from the *FLC* locus in wild type plants and found that AtRRP6L proteins regulate the expression of this transcript. ASL appears to be distinct from the previously described *FLC* antisense transcripts, AS I and II, and our data suggest that it could function in the regulation of levels of H3K27me3 at the *FLC* locus.

### The regulation of lncRNAs could link AtRRP6L functions in development to epigenetic mechanisms

For most exosome complex subunits, mutations cause a lethal phenotype, which indicates that, for most organisms, development requires exosome-mediated regulation of diverse RNAs [8], [61]. Unlike exosome core subunits, RRP6 and AtRRP6-Like proteins are not essential for viability [14], [20]. Most studies of exosome-dependent and exosome-independent RRP6 functions in developmental processes have been performed in fission and budding yeast [18], [21], [24], [49], [57], [62]. In these systems, RRP6 participates in facultative gene silencing and also regulates the transition from mitosis to meiosis through RNA-mediated epigenetic mechanisms [24], [57]. Similar to these findings, we observed that AtRRP6L1 and AtRRP6L2 function in regulation of flowering time by repressing *FLC* expression. The defect in the AtRRP6L proteins results in mis-regulation of antisense RNA production from the *FLC* locus and affects the level of histone modification of the locus.

AS I and II transcripts function in *FLC* silencing in both vernalization and autonomous flowering pathways [37], possibly by affecting the level of H3K4 demethylation [38], [39], although the exact mechanism remains unknown. A decrease in the levels of processed AS I and II in mutants of CstF64 and CstF77, proteins involved in 3′-end processing, leads to increased levels of H3K4 trimethylation and subsequent de-repression of *FLC* [38]. Thus, AtRRP6Ls may contribute to 3′-end processing of the antisense RNAs similarly to the CstFs; indeed, 3′-end processing of various RNAs is one of the well-known functions of the exosome complex. The exosome processes a number of structural RNAs including rRNA, snRNA and snoRNA *via* trimming the 3′-ends of their precursors [63], [64]. Furthermore, RRP6 acts together with the Nrd1-Nab3 termination complex in budding yeast in non-canonical 3′-end processing and termination of the antisense RNA derived from *PHO84*, as well as processing of several other mRNAs [49], [50]. Disruption of the Nrd1-exosome pathway leads to de-repression of reporter genes integrated into heterochromatic regions and results in alteration of chromatin structure at specific loci and heterochromatic regions [11], [21], [22]. The exosome function in processing of mRNA and antisense lncRNA is likely to be conserved in plants, suggesting that AtRRP6L proteins may participate in regulating synthesis of the antisense RNAs derived from the *FLC* locus and this regulation could be important to maintain a repressive chromatin state for silencing of *FLC*.

Surprisingly, we found that the defect of AtRRP6Ls caused a reduction of the level of H3K27 trimethylation, which has not been reported in studies of AS I and II in the vernalization and autonomous flowering pathways [38], [43]. A different intronic sense lncRNA, COLDAIR, derived from intron 1 of *FLC*, physically associates with the PHD-PRC2 complex to establish H3K27 trimethylation during vernalization [40]. Moreover, the AS I and II transcripts are not required for PcG-mediated silencing *via* regulation of H3K27me3 trimethylation in the vernalization pathway [41], [43]. Together, these data suggest that other lncRNAs, not AS I and II, function in regulating H3K27 trimethylation.

AtRRP6Ls might affect the level of H3K27me3 indirectly, by regulating H3K4 demethylation through regulating either AS I and II, or ASL transcripts, *via* a mechanism similar to the interplay between Trithorax and Polycomb groups, which antagonistically regulate the levels of H3K4me3 and H3K27me3 at the *FLC* locus during vernalization [40], [41]. Alternatively, AtRRP6Ls may regulate the level of H3K27me3 directly *via* an unknown mechanism that functions independently of the silencing pathway involving AS I and II. Indeed, ARABIDOPSIS TRITHORAX 1 (ATX1), an *Arabidopsis* homolog of Trithorax 1, dynamically regulates activation of *FLC* through trimethylation but not dimethylation of H3K4 and *atx1* mutations led to the loss of H3K4me3 and gain of H3K27me2 during the vegetative phase, but did not affect H3K4me2 and H3K27me3 [65]. This indicates that the regulation of the levels of H3K4me3 and H3K27me3 at the *FLC* locus could be independent of each other, at least during the vegetative phase. Together, the previous reports and our data suggest that the decrease of H3K27me3 in *rrp6l1/2* mutants could be caused by the decrease in ASL RNA expression, and AtRRP6Ls may participate in the respective pathways for *FLC* silencing through regulating the expression of AS I, AS II, and ASL RNAs.

We identified ASL, a novel, long antisense RNA that is distinct from the previously-described antisense RNAs. We observed that the ASL transcript physically associates with H3K27me3, suggesting that it could play a role in H3K27 trimethylation and function differently than AS I and II RNAs. The PRC2 complex binds ncRNAs with high affinity but does not recognize specific sequences, while its binding affinity correlates with the length of the RNA [66], [67]. Thus, it is possible that ASL transcript could also participate in recruitment of the PRC2 complex to the *FLC* locus, which leads to maintenance of H3K27 trimethylation during vegetative growth. Taken together, the previous reports and our data suggest that the regulation of chromatin structure *via* various lncRNAs is a central mechanism in *FLC* silencing, and different lncRNAs may function in different chromatin modification pathways. The AtRRP6L proteins may play a role in silencing pathways by regulating antisense transcription.

### ASL expression is regulated mostly by AtRRP6L1 and AtRRP6L2

ASL has several features in common with AS I and AS II. First, ASL is transcribed by RNA Pol II; second, it is alternatively spliced, existing in 2 isoforms; third, its transcription is driven by the same promoter that drives AS I and AS II; fourth, the 5′ part of ASL overlaps with the 5′ region of AS I and II. However, in contrast to AS I and II, the ASL transcript is long (over 2,000 nucleotides long), is non-polyadenylated, and extends into intron 1 of *FLC*. These differences suggest that ASL may have functions distinct from the functions of AS I and II in *FLC* silencing, and the mechanism of *FLC* silencing could be more complicated than previously thought.

RNA Pol II transcribes ASL. Various non-polyadenylated Pol II RNAs, such as snRNA, snoRNAs, and some mRNAs, are processed by the exosome, which is recruited by the Nrd1-Nab3-Sen1 termination complex in the noncanonical 3′ end-processing pathway [22], [49], [50], [63], [68]. Thus, the noncanonical 3′ end-processing pathway may also participate in processing of ASL, if this pathway is conserved in plants. However, *Arabidopsis* homologs of Nrd1, Nab3 and Sen1 have not yet been characterized.

The *rrp6l1-3* and *rrp6l2-3* single mutants showed decreased levels of ASL, and ASL was almost undetectable in the *rrp6l1/2* double mutant. Based on the results of the α-amanitin chase experiments, the *rrp6l1-1* mutation does not affect the stability of ASL, suggesting that AtRRP6L1 and AtRRP6L2 could be the main regulators of ASL synthesis. This finding is very intriguing, since defects in the exosome and RRP6 usually lead to abnormal accumulation of various RNAs due to failures of RNA degradation or processing [21], [22], [49], [50], [57]. This result may be caused by an unknown function of AtRRP6L proteins, which participate in either the synthesis or biogenesis of ASL, rather than in its degradation. Indeed, we previously reported that the expression of a number of loci decreased in *AtRRP4* and *AtRRP41* inducible RNAi plants and the *AtCSL4-2* mutant [8], and many of these loci are located within euchromatic regions as well as in regions harboring H3K27me3 (unpublished data). Therefore, the exosome and AtRRP6Ls may function in regulation of RNA synthesis, different from their conventional functions in RNA degradation. Similarly, inactivation of the human homologue of RRP6 leads to dramatically reduced levels of *Xist* ncRNA involved in X-chromosome inactivation, although it remains to be seen whether this effect is direct [69].

In our study, we found that AtRRP6L1 protein physically associates with the ASL transcript, suggesting that AtRRP6L1 plays a direct role in regulation of ASL levels, likely through ASL synthesis rather than degradation. In addition, recent work demonstrated that another 5′-3′ exoribonuclease, Xrn1, also directly contributes to RNA synthesis of several mRNAs in budding yeast, by physically associating with chromatin and contributing to transcription elongation [70]. Alternatively, the decrease in ASL levels in the AtRRP6L mutants may indicate that different RNA decay proteins participate in degradation of these RNAs.

We found that the level of *FLC* transcript was unaffected in the exosome core subunit mutants, *AtRRP4* and *AtRRP41* inducible RNAi lines and *AtCSL4-2* T-DNA mutants, suggesting that the function of AtRRP6L1 and AtRRP6L2 in regulation of *FLC* expression could be independent of the exosome complex. This could also indicate that the exosome core complex is not necessary for metabolism of RNAs produced from the *FLC* locus and different RNA decay factors could participate in their degradation. For example, in yeast, the XRN family of 5′ to 3′ exoribonucleases works in both the nucleus and cytoplasm, and has diverse functions in RNA metabolism [71], including in the degradation of XRN1-sensitive unstable antisense RNAs [72].

### Possible mechanism of AtRRP6L protein function in *FLC* silencing

Silencing of *FLC* is regulated mainly through histone modifications rather than DNA methylation [73]. We previously reported that the exosome complex and AtRRP6L proteins function in DNA methylation-independent silencing and affect the histone modification pathway in some heterochromatic loci in *Arabidopsis* [31]. Along with our previous findings, regulation of *FLC* silencing mainly by histone modifications suggested that the *FLC* locus could be one of the targets of the AtRRP6L proteins. In accord, we observed that defects in AtRRP6L proteins caused a decrease in antisense RNAs, resulting in the alteration of histone modifications and de-repression of *FLC*.

It is intriguing to speculate that AtRRP6L proteins may have dual functions in *FLC* silencing *via* regulation of antisense transcription, which means that AtRRP6L proteins could participate in 2 different pathways, one involved in H3K4 demethylation and the other involved in H3K27 trimethylation. First, AtRRP6Ls could participate in the H3K4 demethylation pathway via regulating synthesis of AS I and AS II. Second, AtRRP6L proteins could function in the H3K27 trimethylation pathway *via* regulating the synthesis of ASL. However, more work will be needed to untangle the interrelationships of the different lncRNAs and the roles they play in the epigenetic architecture at *FLC*. How AtRRP6L proteins and the ncRNAs controlled by them help recruit chromatin modifiers to modulate silencing by affecting histone modifications that repress transcription remains an intriguing topic for future work.

## Materials and Methods

### Plant materials and growth conditions

The *atrrp6l1-3* allele was isolated from the BASTA population from the University of Wisconsin [31]; the *atrrp6l1-1*, *atrrp6l2-2*, *atrrp6l2-3*, *atrrp6l2-4* and *atrrp6l3-1* alleles correspond to SALK_004432, SALK_113786, SALK_011429, and SALK_149898, and SALK_122492, respectively. iRNAi lines of *RRP41*, *csl4-2*, RNA Pol IV (SALK_128428, *nrpd1a-3*, *nrpd1-3*), RNA Pol V (SALK_029919, *nrpd1b-11*, nrpe1-11) mutants were described previously [8], [74], [75]. All Salk alleles are in the Col-0 ecotype and the University of Wisconsin alleles are in the Ws ecotype. The RNAi-mediated knockdown of *RRP41* was induced by germinating and growing seedlings on ½× MS plates containing 8 mM 17β-estradiol, following a previously published method [8].

Long day and short day conditions for plant growth were 16 hours light/8 hours dark and 8 hours light/16 hours dark, respectively. Flowering time was measured by counting rosette leaf number at the time of flowering [76].

### Transgenic lines and vector construction

For chromatin immunoprecipitation (see below), we used the Tandem Affinity Purification (TAP) affinity tag to selectively precipitate RRP6L1 by expressing a RRP6L1- TAP fusion protein. For construction of plant lines with affinity-tagged *RRP6L1* for RNA-IP and ChIP, we complemented *atrrp6l1-3* with the *RRP6L1-TAP* transgene. For RRP6L1-TAP complementation, the entire genomic region of *RRP6L1* including 1.5 kb upstream from the ATG codon was amplified by PCR using LA *taq* polymerase (Takara) and cloned into TAP-tag carrying destination vector pDB1008 [8]. For complementation with the TAP tagged transgene, the homozygous *atrrp6l1-3* mutant was transformed using *Agrobacterium*-mediated transformation and the progeny plants containing both the T-DNA insertion allele and the transgene were identified by PCR.

### RNA analysis

Trizol reagent (Invitrogen) was used to isolate total RNA from seedlings. 10-, 14-, 18-, and 21-day-old seedlings were used for examining the expression of ASL. 11-day-old seedlings were used for investigating expression of genes and antisense RNAs examined in our study. For RT-qPCR, 2–4 µg of total RNA was digested with DNase I (Fermentas) and reverse transcribed for one hour at 42°C (oligo-dT primers) or at 50°C (gene-specific primers), with 100 units of PrimeScript reverse transcriptase (Takara). RT-qPCR (MyiQ-iCycler; Bio-Rad) was used to quantify transcripts using the comparative threshold cycle method (ΔΔC_t_, Table S1 shows primer sequences), with *ACTIN 7* (At5g09810) as an internal reference.

For tiling RT-PCR, sets of serial primers were designed in intervals of 100–200 nt. After obtaining the full-length ASL transcript, another set of overlapping primers was designed to make sure the 3′ and 5′-ends of the RNA have been isolated. The PCR products amplified by tiling RT-PCR were cloned into the pBluescript KS vector and sequenced using T7 and T3 primers.

### Chromatin Immunoprecipitation (ChIP) assays

ChIP was conducted following a previously-described method [77]. Each experiment used 1.5 grams of tissue from 11-day-old seedlings. All ChIP experiments used at least two biological replicates and at least two technical replicates. Anti-H3K4me3 (07-473) and anti-H3K27me3 (ab6002) were purchased from Millipore and Abcam, respectively. IgG Sepharose 6 Fast Flow (GE Healthcare) was used for ChIP using RRP6L1-TAP tagged line. The mock antibody control used an equal amount of chromatin that was not treated with antibody. The ChIPed DNA was purified using PCR purification kit (Fermentas) and qPCR was performed. Supplemental Table S1 lists the primers used for PCR.

### Micrococcal nuclease (MNase) ChIP

MNase-ChIP was performed following a previously-described method [78]. Two grams of tissue from 11-day-old seedlings was fixed using 1% formaldehyde solution for 10 min and washed with distilled water several times. The fixed samples were homogenized with HONDA buffer (20 mM HEPES-KOH pH 7.4, 0.44 M sucrose, 1.25% Ficoll, 2.5% Dextran T40, 10 mM MgCl_2_, 0.5% Triton X-100, 5 mM DTT, 1 mM PMSF, 1% plant protease inhibitors) and then filtered through miracloth. After isolation of the nucleus-containing fraction by centrifugation, the fraction was treated with MNase (NEB) at 37°C for 10 min. Anti-histone H3 (ab1791) was used for the ChIP. The purification of ChIPed DNA and qPCR was performed as described in ChIP assay.

### RNA Immunoprecipitation (RNA-IP)

RNA-IP assays were performed as described previously [31], [79]. Two grams of tissue from seedlings at 11-days-old was fixed with 1% formaldehyde. For purification of RRP6L1-TAP or RRP6L1 RNA complexes, the chromatin was incubated with prewashed IgG Sepharose 6 Fast Flow (GE Healthcare) or with polyclonal anti-AtRRP6L1 antibodies, respectively, at 4°C overnight. H3K27me3-RNA complex purification was performed using anti-H3K27me3 (ab6002) overnight following by incubation with protein A agarose beads. Immunoprecipitated RNA purification used phenol∶chloroform and PrimeScript reverse transcriptase (Takara) and sequence specific primers were used for cDNA synthesis. Supplemental Table S1 lists the primers used for PCR.

### Alpha-amanitin treatment

Eleven-day-old seedlings were treated with 5 µM α-amanitin (Sigma) for 0, 6 and 9 h or 17 h. After RNA extraction, cDNA was synthesized using ASL-specific and *ACTIN 7* primers, followed by qRT-PCR. The level of ASL transcript was normalized relative to the level of *ACTIN 7* transcript.

### Terminator 5′-Phosphate-Dependent Exonuclease (TPE) treatment

Total RNA extracted from 11-day-old seedlings was treated with TPE (Epicentre) at 42°C, and purified with phenol∶chloroform. Complementary cDNA was synthesized using RNA sequence specific primers followed by RT-qPCR.

## Supporting Information

Figure S1 Expression of *FLC* and AS I and II in different *rrp6l1* and *rrp6l2* mutants, and ChIP assay of *rrp6l1-3* using anti-H3K27me3. (A) The late-flowering phenotype and RT-PCR analysis of *FLC* mRNA expression of *rrp6l1-1/rrp6l2-2/rp6l3-1* mutants grown under long day conditions. 20-day-old (after transferring to soil) plants are shown. RRP6L3 is the cytoplasmic protein [20] and thus is very unlikely to contribute to the late flowering phenotype through derepressing FLC. (B) RT-PCR analysis of *FLC* mRNA expression in *rrp6l1-3*, *rrp6l1-1*, *rrp6l2-3*, and *rrp6l2-4* single mutant alleles. (C) RT-qPCR analysis of AS I and AS II transcripts in *rrp6l1-3* and *rrp6l2-3* single mutants. Ws and Col-0 ecotypes, were used as wild-type controls. The expression of AS I and II transcripts was normalized to the expression of total antisense RNA, as described previously [38]. (D) The level of H3K27me3 in *rrp6l1-3* mutants. ChIP assay was performed using H3K27me3 antibodies. The level of H3K27me3 in *rrp6l1-3* mutants was plotted relative to the level of H3K27me3 in Col-0 plants. The error bars in ChIP experiments represent the standard error of the mean and correspond to the difference between 2 biological replicates.
(TIF)

Figure S2 Tiling RT-PCR to identify full-length ASL RNA and examine ASL expression. (A) The strategy of tiling RT-PCR to obtain full-length ASL. Arrowheads indicate serial primers used in RT-PCR. PCR products obtained after tiling RT-PCR were cloned for sequencing analysis. (B) Expression of ASL transcript in *nrpd1* and *nrpe1* mutants. *TUBULIN 2* was used as the loading control. (C) RT-PCR of ASL in *rrp6l1-1* and *rrp6l2-4* mutants. The ASL transcript was normalized to *ACTIN 7*.
(TIF)

Table S1 Oligonucleotides used in this study.
(XLS)
