# Supplementary figures and images for: Arabidopsis RRP6L1 and RRP6L2 Function in FLOWERING LOCUS C Silencing via Regulation of Antisense RNA Synthesis
Source: PLoS Genet. 2014 Sep 11;10(9):e1004612. doi: 10.1371/journal.pgen.1004612 (PMC4161302; doi:10.1371/journal.pgen.1004612)

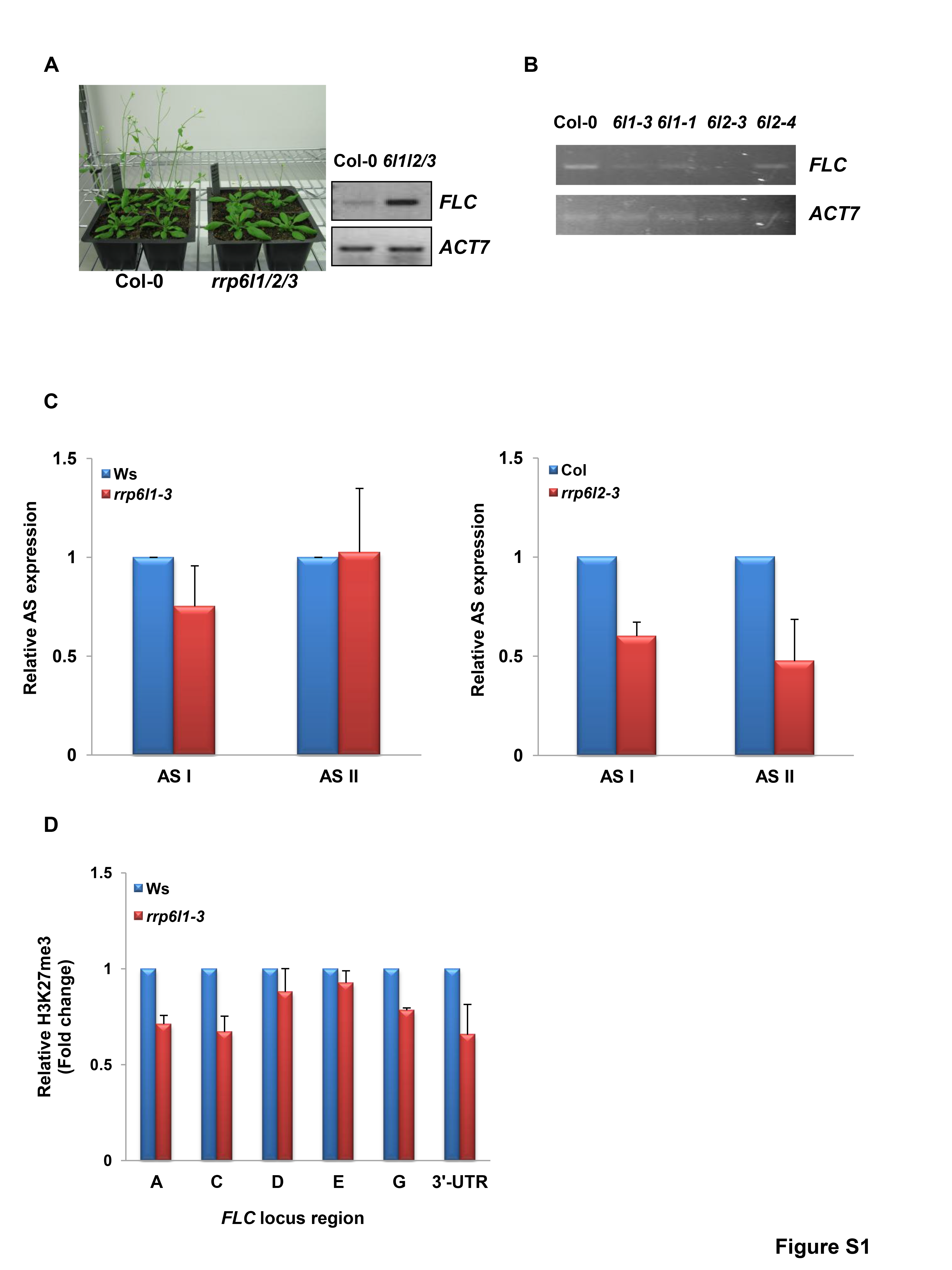

Supplement: Figure S1 — Expression of FLC and AS I and II in different rrp6l1 and rrp6l2 mutants, and ChIP assay of rrp6l1-3 using anti-H3K27me3. (A) The late-flowering phenotype and RT-PCR analysis of FLC mRNA expression of rrp6l1-1/rrp6l2-2/rp6l3-1 mutants grown under long day conditions. 20-day-old (after transferring to soil) plants are shown. RRP6L3 is the cytoplasmic protein [20] and thus is very unlikely to contribute to the late flowering phenotype through derepressing FLC. (B) RT-PCR analysis of FLC mRNA expression in rrp6l1-3, rrp6l1-1, rrp6l2-3, and rrp6l2-4 single mutant alleles. (C) RT-qPCR analysis of AS I and AS II transcripts in rrp6l1-3 and rrp6l2-3 single mutants. Ws and Col-0 ecotypes, were used as wild-type controls. The expression of AS I and II transcripts was normalized to the expression of total antisense RNA, as described previously [38]. (D) The level of H3K27me3 in rrp6l1-3 mutants. ChIP assay was performed using H3K27me3 antibodies. The level of H3K27me3 in rrp6l1-3 mutants was plotted relative to the level of H3K27me3 in Col-0 plants. The error bars in ChIP experiments represent the standard error of the mean and correspond to the difference between 2 biological replicates. (TIF) [file pgen.1004612.s001.tif]

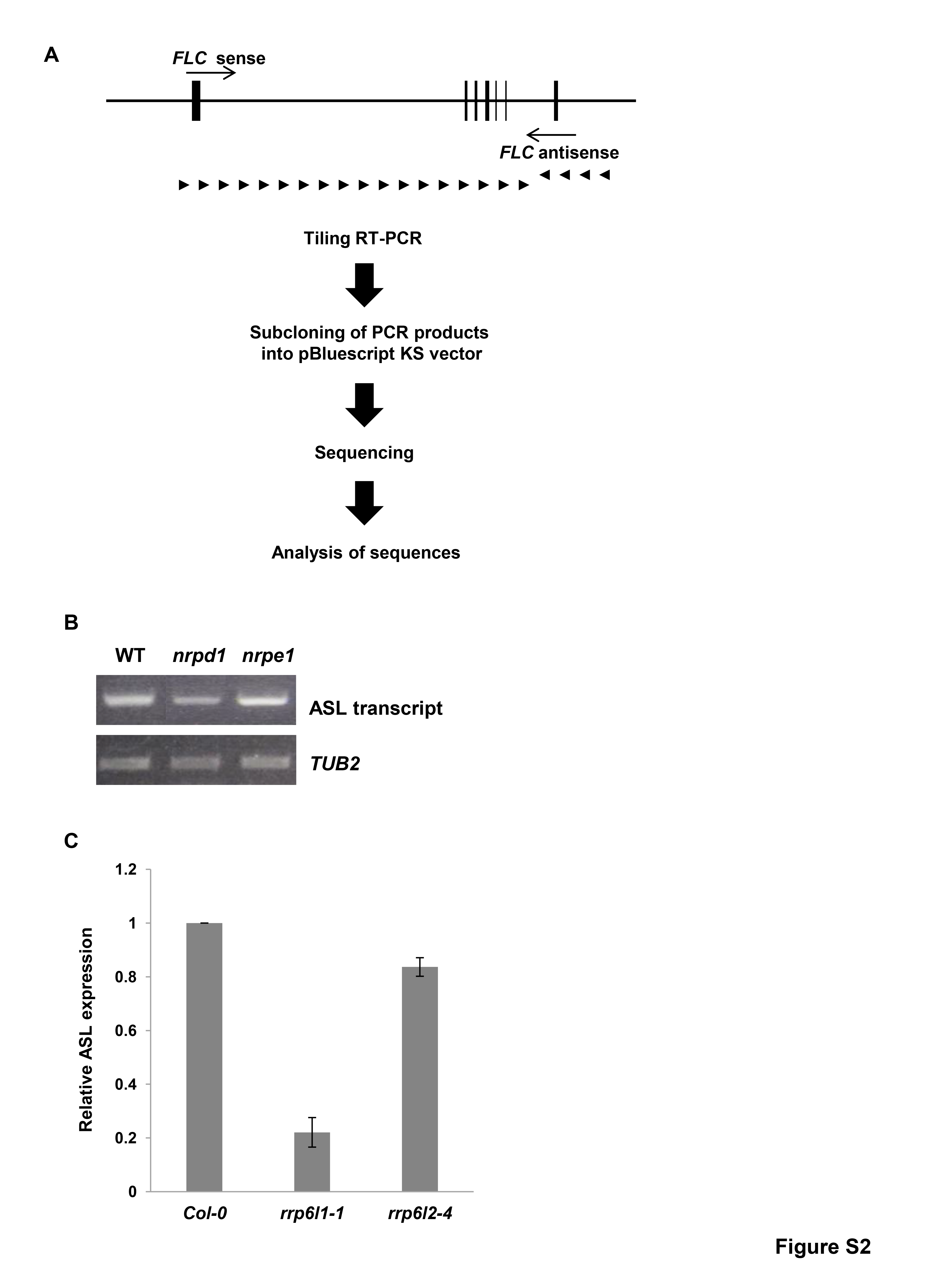

Supplement: Figure S2 — Tiling RT-PCR to identify full-length ASL RNA and examine ASL expression. (A) The strategy of tiling RT-PCR to obtain full-length ASL. Arrowheads indicate serial primers used in RT-PCR. PCR products obtained after tiling RT-PCR were cloned for sequencing analysis. (B) Expression of ASL transcript in nrpd1 and nrpe1 mutants. TUBULIN 2 was used as the loading control. (C) RT-PCR of ASL in rrp6l1-1 and rrp6l2-4 mutants. The ASL transcript was normalized to ACTIN 7. (TIF) [file pgen.1004612.s002.tif]
